# IL-1beta Signals through the EGF Receptor and Activates Egr-1 through MMP-ADAM

**DOI:** 10.1371/journal.pone.0039811

**Published:** 2012-07-06

**Authors:** Estella Sanchez-Guerrero, Elya Chen, Maaike Kockx, Si-Wei An, Beng H. Chong, Levon M. Khachigian

**Affiliations:** Centre for Vascular Research, University of New South Wales, Sydney, Australia; Maastricht University, Netherlands

## Abstract

The immediate-early gene Egr-1 controls the inducible expression of many genes implicated in the pathogenesis of a range of vascular disorders, yet our understanding of the mechanisms controlling the rapid expression of this prototypic zinc finger transcription factor is poor. Here we show that Egr-1 expression induced by IL-1beta is dependent on metalloproteinases (MMP) and a disintegrin and a metalloproteinase (ADAM). Pharmacologic MMP/ADAM inhibitors and siRNA knockdown prevent IL-1beta induction of Egr-1. Further, IL-1beta activates Egr-1 via the epidermal growth factor receptor (EGFR). This is blocked by EGFR tyrosine kinase inhibition and EGFR knockdown. IL-1beta induction of Egr-1 expression is reduced in murine embryonic fibroblasts (mEFs) deficient in ADAM17 despite unbiased expression of EGFR and IL-1RI in ADAM17-deficient and wild-type mEFs. Finally, we show that IL-1beta-inducible wound repair after mechanical injury requires both EGFR and MMP/ADAM. This study reports for the first time that Egr-1 induction by IL-1beta involves EGFR and MMP/ADAM-dependent EGFR phosphorylation.

## Introduction

Intimal hyperplasia is a key feature in the pathogenesis of atherosclerosis, restenosis following percutaneous coronary intervention, and bypass graft stenosis. Vascular smooth muscle cell (SMC) proliferation, migration, extracellular matrix deposition and inflammatory cell infiltration contribute to neointima formation [1]. In response to vascular injury, normally contractile SMCs undergo de-differentiation to a proliferative and migratory state, the so-called synthetic phenotype [2] driven by local environmental cues [3]. The immediate-early gene and zinc finger transcription factor, early growth response-1 (Egr-1) [4] is poorly expressed in the artery wall and induced by vascular injury [5]. Egr-1 is stimulated by cytokines, growth factors, hypoxia, oxidized lipoprotein, shear stress, angiotensin II (Ang II) and other injurious stimuli [6]. Once activated Egr-1 mediates a series of transcriptional changes that lead to altered expression of key genes, such as the platelet-derived growth factors (PDGF), transforming growth factor-beta1 (TGF-beta1), matrix metalloproteinases (MMPs), tissue factor (TF) and heparanase [5], [7]–[9]. Egr-1 transcription is dependent on Ras-Raf-MEK-ERK1/2 signaling and multiple serum response elements in the Egr-1 promoter [10]. Recent studies by our group demonstrate that Egr-1 regulates its own transcription [10]. Moreover, Egr-1 induction by the pro-inflammatory cytokine interleukin-1beta (IL-1beta) [11] involves the MEK-ERK1/2 and MSK pathway, and the phosphorylation and acetylation of histone H3 [10]. IL-1beta has been implicated in the process of neointima formation [12] through its mitogenic effects on SMCs [13], [14]. IL-1RI null mice exhibit attenuated intimal hyperplasia following artery ligation [15]. Mice lacking IL-1 receptor antagonist (IL-1ra) exhibit enhanced neointima formation following femoral artery injury [16].

Egr-1 plays a pivotal role as a mediator of SMC growth and intimal thickening in the restorative response to vascular injury. Egr-1 is expressed in human and animal models of atherosclerosis [17]. Furthermore, Egr-1 inhibition by catalytic DNA blocks SMC replication and regrowth after *in vitro* scraping injury, and prevents intimal thickening after balloon injury in rats [18], permanent ligation in rat carotid arteries [19] and stenting in porcine coronary arteries [20]. Decoy oligonucleotides targeting Egr-1 inhibit intimal hyperplasia after balloon injury in rabbits [21]. Egr-1 is thus key in the pathogenesis of vascular disorders, yet our understanding of the mechanisms controlling its expression is poor.

Extracellular proteases, such as MMPs and plasminogen activators are induced during vascular injury. These contribute to both neointima formation and plaque instability by degrading matrix and non-matrix substrates [22] and their production is regulated by cytokines and growth factors. Active MMPs are produced from pro-MMP by the local action of proteases [23]. Once activated, MMPs participate in a diverse range of cellular processes including cell proliferation, migration and matrix remodeling [24]. MMPs and a disintegrin and a metalloproteinase (ADAM)s cleave latent growth factors, whereby cleaved active ligand, in turn, binds and activates its receptor [23]. MMPs [25], [26] and ADAM17 [27] mediate neointima formation in models of arterial injury. A prototypic example of MMP/ADAM-dependent shedding is epidermal growth factor receptor (EGFR) activation. The EGFR family consists of four transmembrane receptors that include EGFR (ErbB1 or HER1), ErbB2 (HER2, Neu), ErbB3 (HER3), and ErbB4 (HER4) [28], [29]. The EGFR also known as ErbB1 or HER1 is a 170 kDa transmembrane glycoprotein characterised by an extracellular ligand-binding domain with two cysteine-rich regions, a single α-helical transmembrane domain and a cytoplasmic domain which contains the tyrosine kinase region [30]. The tyrosine kinase region is followed by a carboxy-terminal tail, which harbors the autophoshorylation sites. Importantly, this domain is well conserved within the EGFR family except in ErbB3 in which some amino acids are changed, resulting in impaired tyrosine kinase activity [31]. Pathways demonstrating a role for MMP/ADAM in EGF ligand shedding by G protein-coupled receptors (GPCR) is termed EGFR transactivation or the “triple membrane-passing signaling” paradigm [32]. Here we report MMP/ADAM(17)-dependent activation of EGFR by IL-1beta that results in the induction of Egr-1.

## Materials and Methods

### Chemicals

Human recombinant IL-1beta was purchased from Calbiochem (Darmstad, Germany). MMP inhibitors (TAPI-1, GM6001+, GM6001-) and EGFR inhibitors were purchased from Calbiochem. Rabbit polyclonal antibodies to EGFR and IL-1R1, goat polyclonal antibodies to ADAM17 and mouse monoclonal antibodies to phospho-EGFR (Tyr^845^) were obtained from Santa Cruz Biotechnology (Santa Cruz, CA, USA). Mouse monoclonal antibodies to beta-actin were obtained from Sigma (St Louis, MO, USA). Rabbit monoclonal antibodies Egr-1 were obtained from Cell Signaling (Danvers, MA, USA).

### Cell Culture

WKY12-22 pup rat aortic SMCs were obtained as a gift from Dr Stephen Schwartz, University of Washington [8], [33] and cultured in Waymouth’s medium (Sigma), pH 7.4, with antibiotics [34] and 10% fetal bovine serum (FBS) in an Air Jacket CO_2_ incubator at 5% CO_2_ and 37°C. SMCs were rendered growth-quiescent by incubation in serum-free medium for 24 h prior to the addition of inhibitors. In MMP, EGFR inhibitor studies, SMCs were incubated with GM6001+ (25 µM), GM inactive analogue GM6001- (25 µM), TAPI-1 (10 µM), AG1478 (5 µM), PD153035 (5 µM) for 30 min. Cells were stimulated with 10 ng/ml IL-1beta for 30 min prior to mRNA and protein isolation. Wild type and ADAM17-deficient mEFs were grown on gelatin-coated 6 well plates, in high glucose DMEM (Gibco, Carlsbad, CA, USA), supplemented with 10 units/ml penicillin, 10 µg/ml streptomycin, 10% FBS with L-glutamine in an Air Jacket CO_2_ incubator at 5% CO_2_ and 37°C.

### Total RNA Preparation and Reverse Transcriptase Reaction

Cells were washed twice with cold PBS and total RNA was extracted with TriReagent® (Sigma). cDNA was synthesized from 5 µg of RNA using the Super Script II First Strand Synthesis Kit (Invitrogen, Carlsbad, CA, USA) as per manufacturer’s instructions. cDNA was stored at –20°C until use.

### Real-time PCR

Real-time quantitative PCR was performed using ABI PRISM7700 Sequence Detection System in a final volume of 20 µl containing 1 µl of cDNA, 12.5 µl of SYBR Green Master Mix (Applied Biosystems, Carlsbad, CA, USA), 0.5 µM of forward and reverse primers (Sigma) in DNAse-free water at the following PCR conditions: (rat amplicons) 50°C for 2 min then 94°C for 10 min, and 40 cycles at 94°C for 20 sec, 60°C for 45 sec and 72°C for 20 sec; (mouse amplicons) 50°C for 2 min then 94°C for 10 min, and 40 cycles at 94°C for 30 sec, 62°C for 30 sec and 72°C for 20 sec.

Primer sequences were:

Egr-1 (rat) were (forward) 5′-GCC TTT TGC CTG TGA CAT TT-3′, (reverse) 5′-AGC CCG GAG AGG AGT AAG AG-3′.

Beta-actin (rat) were (forward) 5′-AGCCATGTACGTAGCCATCC-3′, (reverse) 5′-CTC TCA GCT GTG GTG GTG AA-3′.

Egr-1 (mouse) were (forward) 5′-GAG CGA ACA ACC CCT ATG AGC-3′, (reverse) 5′-AGG CCA CTG ACT AGG CTG AA-3′.

GAPDH (mouse) were (forward) 5′-ACC ACA GTC CAT GCC ATC AC-3′, (reverse) 5′-TCC ACC ACC CTG TTG CTG TA-3′.

Primer product size was verified on 2% agarose/TBE gels.

### Western Blot Analysis

SMCs were grown in 100 mm petri dishes, and mEFs were grown on gelatin-coated 6 well plates and rendered growth quiescent by serum-deprivation for 24 h. Cell lysates were prepared as described [35]. Protein estimation was estimated with BCA protein assay kit (Pierce, Rockford, IL, USA). Total cell lysates (10–40 µg) were resolved by 6% or 10% SDS-polyacrylamide gel electrophoresis and transferred onto Immobilon-P transfer membranes (Millipore, Billerca, MA, USA). Membranes were blocked for 1 h with 5% skim milk in 1% PBS, 0.05% Tween 20 at 22°C. Membranes were washed followed by incubation overnight with primary antibodies. After three more washes, IgG-conjugated with horseradish peroxidase (HRP) secondary antibodies were incubated for 1 h at 22°C with the membranes. Subsequently, membranes were incubated with chemiluminescence (Perkin Elmer Life Sciences, Shelton, CT, USA) for 1 min then exposed the film for 1–20 min. Identical samples were occasionally run on different gels for improved band separation and clarity.

### Small Interfering RNA (siRNA) Studies

Growth-quiescent SMCs in 100 mm petri dishes were transfected with 0.1 µM siRNA targeting rat EGFR, ADAM17 or ErbB4 using DOTAP/DOPE (Avanti Polar Lipids, Alabaster, AL, USA) or Lipofectamine (Life Technologies, Carlsbad, CA, USA). After 20 h, the cells were stimulated with 10 ng/ml IL-1beta for 30 min. Total cell lysates were collected. siRNA-ON-TARGETplus and the ON-TARGETplus non-specific siRNA were purchased from Dharmacon Technologies (Lafayette, CO, USA).

### Iodination of IL-1beta and Binding of ^125^I-IL-1beta to ADAM17WT and ADAM17-deficient mEFs

Recombinant IL-1beta (5 µg) was radioiodinated with Iodination Beads (Pierce Biotechnology, Rockford, IL, USA) using N-chloro-benzenesulfonamide (sodium salt) immobilized on nonporous polystyrene beads. Growth-quiescent mEFs in 24 well plates (1.8×10^4^ cells/well) were washed and incubated with ice-cold 1% BSA/PBS for 1 h at 4°C. The cells were incubated with increasing amounts of ^125^I-IL-1beta (1×10^2^ cpm/well to 3×10^6^ cpm/well) in 1% BSA/PBS for a further 1 h at 4°C. The cells were washed with 1% BSA/PBS at 4°C and lysed with 1M NaOH prior to assessment of counts in an automated gamma-counter.

**Figure 1:**
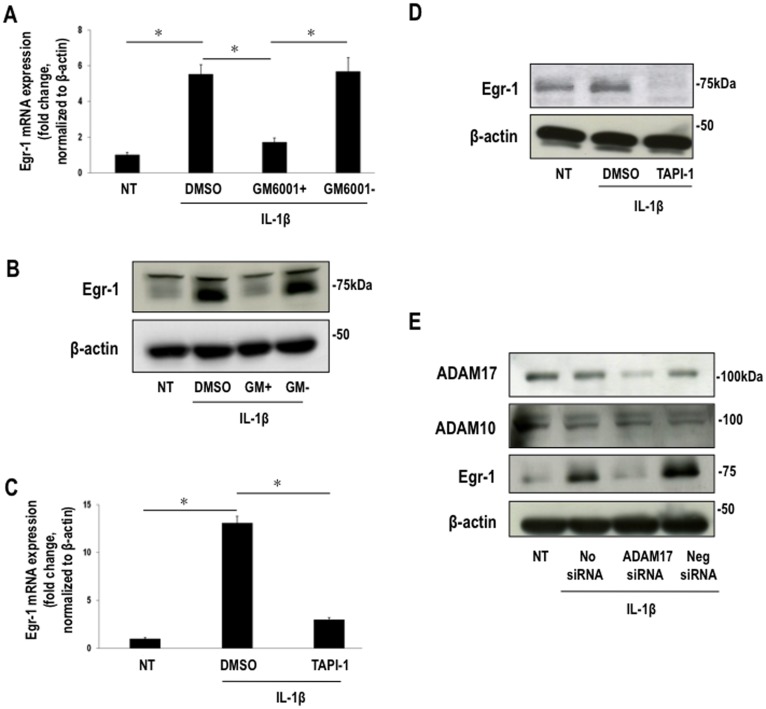
IL-1beta-inducible Egr-1 expression is MMP/ADAM-dependent. Growth-quiescent SMCs were treated with MMP inhibitor GM6001+ (25 µM) and TAPI-1 (10 µM) for 30 min before stimulation with IL-1beta (10 ng/ml) for another 30 min (unless indicated otherwise). (**A**) Egr-1 mRNA levels in GM6001+ or GM6001- treated SMCs treated with IL-1beta by qPCR. Data were normalized to beta-actin. (**B**) Western blotting for Egr-1 or beta-actin in total extracts of SMCs treated with GM6001+ or GM6001-. (**C**) Egr-1 mRNA levels in TAPI-1 treated SMCs treated with IL-1beta by qPCR. Data were normalized to beta-actin. (**D**) Western blotting for Egr-1 or beta-actin of total extracts of SMCs treated with TAPI-1. DMSO was used as a carrier. (**E**) Western blotting for ADAM17, ADAM10, Egr-1 or beta-actin of total extracts of SMCs transfected overnight with ADAM17 siRNA (0.1 µM) and treated with IL-1beta for 30 min.

### Statistical Analysis

Data was analysed by one-way ANOVA, with Bonferroni’s Multiple Comparison Test. * indicates *p*<0.05. ns indicates no statistical difference.

### In vitro Mechanical Injury

SMCs were grown in 6-well plates to 60% confluence in Waymouth’s medium containing 10% FBS. The cells were rendered quiescent by culturing in serum-free Waymouth’s medium for 24 h. The monolayers were scratched using a P20 micropipette tip. The medium was replaced to remove cell debris. Inhibitors were added to the cells for 30 min followed by exposure to IL-1beta (10 ng/ml). Cell growth in the denuded zone was monitored and photographs at 100x magnification were taken at 24 h after injury.

### Densitometry

Band intensity was quantitated using the Gel Analysis method in the NIH ImageJ program. Data was plotted as relative intensity of treatment versus non-treatment.

**Figure 2:**
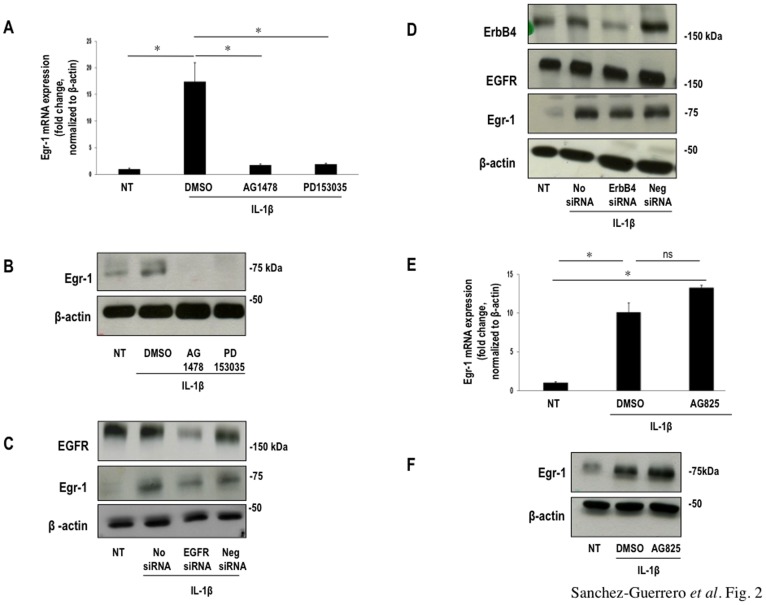
Induction of Egr-1 by IL-1beta is EGFR-dependent. Growth-quiescent SMCs were incubated with EGFR inhibitors AG1478 (5 µM) and PD153035 (5 µM) for 30 min, and exposed to IL-1beta (10 ng/ml) for another 30 min (unless indicated otherwise). (**A**) Egr-1 mRNA levels in AG1478- and PD153035-treated SMCs incubated with IL-1beta by qPCR. Data were normalized to beta-actin. (**B**) Western blotting for Egr-1 or beta-actin of total extracts of SMCs treated with AG1478 or PD153035. (**C**) Western blotting for EGFR, Egr-1 or beta-actin of total extracts of SMCs transfected overnight with EGFR siRNA (0.1 µM) and treated with IL-1beta for 30 min. (**D**) Western blotting for ErbB4, EGFR, Egr-1 or beta-actin of total extracts of SMCs transfected overnight with ErbB4 siRNA (0.1 µM) and treated with IL-1beta for 30 min. (**E**) Egr-1 mRNA levels in AG825 (10 µM for 30 min)-treated SMCs incubated with IL-1beta for 30 min by qPCR. Data were normalized to beta-actin. (**F**) Western blotting for Egr-1 or beta-actin in total extracts of SMCs pretreated with AG825 (10 µM for 30 min) then stimulated with IL-1beta for another 30 min.

**Figure 3:**
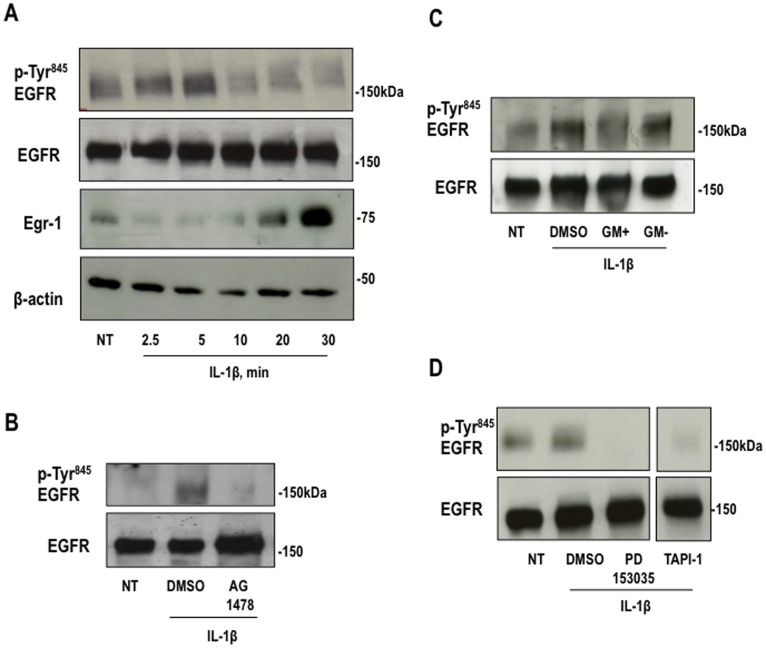
IL-1beta trans-activates EGFR in a time-dependent manner and it is dependent on MMP activity. (**A**) Growth-quiescent SMCs were stimulated with IL-1beta (10 ng/ml) for the indicated times. Total protein extracts were resolved by SDS-PAGE and subjected to Western blot analysis using antibodies for Egr-1, EGFR phospho-Tyr^845^, total EGFR and beta-actin. Alternatively, Western blotting for EGFR phospho-Tyr^845^ or EGFR of total extracts of SMCs treated with (**B**) AG1478 (5 µM), (**C**) GM6001+ or GM6001- (25 µM) (**D**) TAPI-1 (10 µM), PD153035 (5 µM) for 30 min followed by IL-1beta stimulation for 5 min.

**Figure 4:**
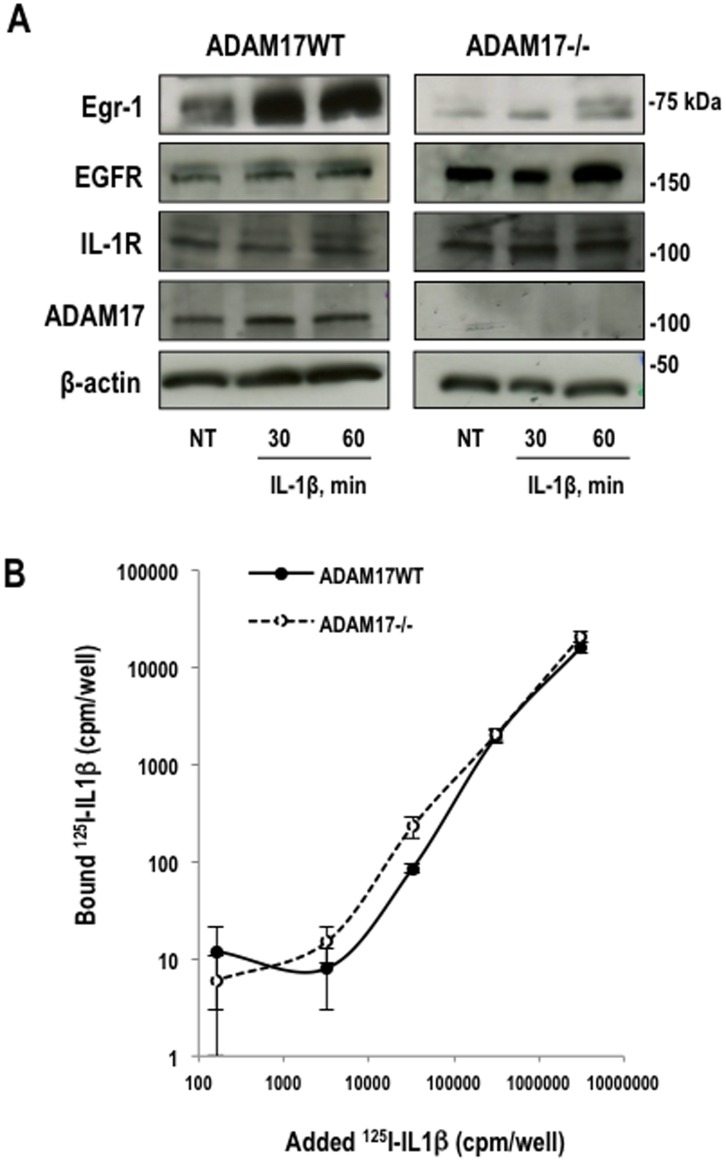
ADAM17 is required for IL-1beta–inducible Egr-1 expression. (**A**) Western blotting for Egr-1, ADAM17, EGFR, IL-1RI or beta-actin using total extracts of growth-quiescent wild-type or ADAM17-deficient mEFs treated with IL-1beta for 30 or 60 min. (**B**) Interaction of ^125^I-IL-1beta with ADAM17WT and ADAM17-deficient cells. Growth-quiescent ADAM17WT and ADAM17−/− mEFs (1.8×10^4^ cells/well) were incubated with increasing amounts of ^125^I-IL-1beta in 1% BSA/PBS for 1 h at 4°C. The cells were washed and lysed with 1M NaOH prior to assessment of counts in an automated gamma-counter.

**Figure 5:**
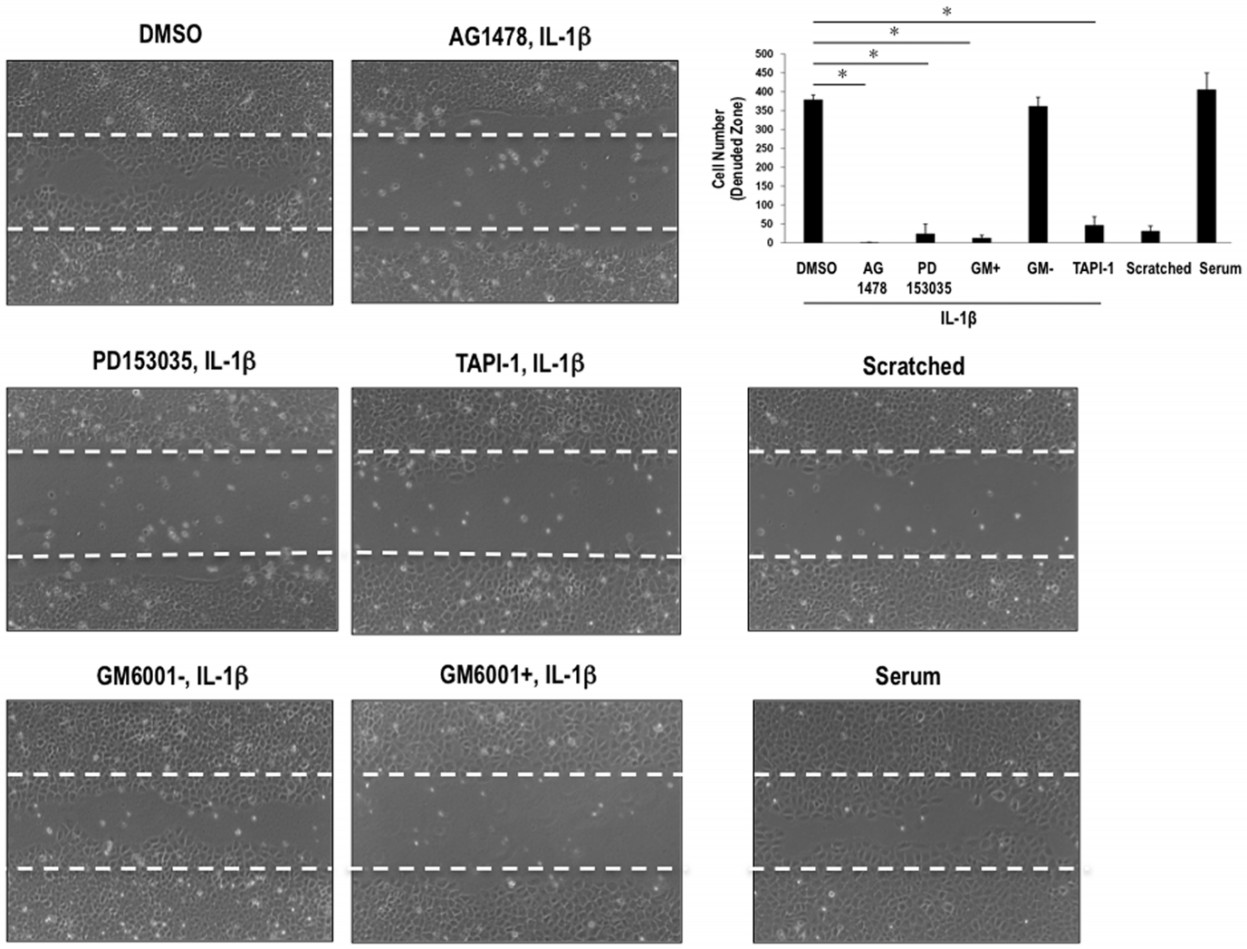
MMP and EGFR inhibitors block SMC wound repair. Growth-quiescent SMCs were incubated with GM6001+ or GM6001- (25 µM), TAPI-1 (10 µM), AG1478 (5 µM), PD153035 (5 µM) or vehicle for 30 min followed by *in vitro* scraping injury and addition of IL-1beta (10 ng/ml). Photographs of the denuded zone were taken at 24 h.

## Results

Our previous studies indicate that Egr-1 plays a key regulatory role in the inducible expression of many genes implicated in a variety of vascular pathologic processes [7], [10], [18], [35]–[37]. The induction of Egr-1 is a rapid and transient process, with IL-1beta increasing Egr-1 mRNA within 1 h [11]. Given the broad pathobiologic importance of Egr-1, we hypothesized that an indirect mechanism, that involves receptor crosstalk, may mediate cytokine induction of this key transcription factor.

We pretreated growth-quiescent SMCs with a series of pharmacologic MMP/ADAM inhibitors, including GM6001+ (a pan spectrum MMP inhibitor) [38] and TAPI-1 (an ADAM17-specific inhibitor) [39] and exposed the cells to IL-1beta for a further 30 min. GM6001+ (**Figs. 1A & B** and **Fig. S1A**) and TAPI-1 (**Figs. 1C & D** and **Fig. S1B**) almost completely inhibited IL-1beta-inducible Egr-1 mRNA and protein expression. In contrast GM6001-, the inactive counterpart of GM6001+ had no effect (**Figs. 1A & B** and **Fig. S1A**). Small interfering RNA (siRNA) [40] reduced ADAM17 expression (**Fig. 1E** and **Fig. S1C**) and blocked IL-1beta-inducible Egr-1 expression as compared with the negative siRNA control. In contrast, the siRNA had no effect on levels of ADAM10 **(** **Fig. 1E** and **Fig. S1C**). These data show that Egr-1 induction by IL-1beta is MMP/ADAM-dependent.

We next determined whether IL-1beta-inducible Egr-1 expression involved EGFR and used specific inhibitors of EGFR intrinsic kinase activity, the tyrphostin AG1478 [41] and PD153035 [41]. Pretreatment of quiescent SMCs with these inhibitors caused inhibition of IL-1beta-inducible Egr-1 mRNA (**Fig. 2A** **)** and protein expression **(** **Fig. 2B** and **Fig. S2A**). AG1478 and PD153035 prevented Egr-1 expression at nanomolar concentrations (**Figs. S2B** & **C**). To provide further evidence of a role for EGFR in IL-1beta-inducible Egr-1 expression, we transfected SMCs with EGFR siRNA. This knocked down EGFR levels compared with cells transfected with a negative siRNA control (**Fig. 2C** and **Fig. S2D**). It also suppressed IL-1beta-inducible Egr-1 protein expression (**Fig. 2C** and **Fig. S2D**). These data demonstrate that IL-1beta induction of Egr-1 in SMCs is EGFR-dependent.

To determine whether IL-1beta-inducible Egr-1 expression is directly attributable to EGFR type 1 inhibition rather than blockade of another member of the EGFR family, we transfected SMCs with siRNA to ErbB4. ErbB4 silencing, which was confirmed by Western blotting had no effect on IL-1beta-inducible Egr-1 expression (**Fig. 2D** and **Fig. S2E**). IL-1beta-inducible Egr-1 expression is thus ErbB4-independent. Moreover, Egr-1 induction by IL-1beta was not affected by the ErbB2 inhibitor AG825 [42] (**Fig. 2E**).

We next demonstrated the MMP/ADAM-dependency of IL-1beta-inducible EGFR activation by using GM6001+ and TAPI-1 in experiments assessing EGFR phosphorylation at Tyr^845^. IL-1beta stimulated Tyr^845^ phosphorylation in a transient manner and optimally within 5 min (**Fig. 3A** and **Fig. S3A**). Egr-1 is not expressed until after this time (**Fig. 3A** and **Fig. S3A**). **Figs. 3B-D** and the corresponding band quantitation (**Figs. S3B-D**) revealed the effect of IL-1β on EGFR phosphorylation relative to total (non-phospho-specific) EGFR. AG1478 blocked EGFR phosphorylation at this time confirming inhibition of intrinsic EGFR kinase activity (**Fig. 3B** and **Fig. S3B**). GM6001+ and TAPI-1 each also inhibited IL-1beta-inducible EGFR phosphorylation, whereas GM6001- failed to inhibit (**Figs. 3C & D** and **Figs. S3C** & **D**). PD153035 inhibited IL-1beta activation of EGFR Tyr^845^ (**Fig. 3D** and **Fig. S3D**). These results demonstrate that IL-1beta transactivates the EGFR and does so in an MMP/ADAM-dependent manner.

The preceding data support a key role for EGFR in the induction of Egr-1 in SMCs exposed to IL-1beta. Inhibition by TAPI-1 suggested the involvement of ADAM17 in this process. We interrogated the role of ADAM17 in IL-1beta–inducible Egr-1 expression in more precise terms using mouse embryonic fibroblasts (mEFs) deficient in ADAM17. Western blotting confirmed ADAM17 deficiency in ADAM17^−/−^ cells (**Fig. 4A** and **Fig. S4**). IL-1beta induced Egr-1 protein expression in wild-type mEFs in a time-dependent manner (**Fig. 4A** and **Fig. S4**). In contrast, Egr-1 was poorly induced by IL-1beta in ADAM17-deficient cells (**Fig. 4A** and **Fig. S4**) despite there being no difference in the capacity of ^125^I-IL-1beta to bind ADAM17WT and ADAM17-deficient cells (**Fig. 4B**), and the unbiased expression of EGFR and IL-1RI in both cell types (**Fig. 4A** and **Fig. S4**).

Metalloproteinases play a pivotal role in the process of cell migration and wound repair. For example in SMCs, MMP-inhibition blocks Ang II-induced migration and proliferation [43]. We injured SMCs by scraping *in vitro* and observed the reparative response in the presence of IL-1beta to investigate the effect of TAPI-1 on wound repair stimulated by IL-1beta (**Fig. 5**). This reparative response was inhibited by TAPI-1 (**Fig. 5**). It was also inhibited by GM6001+ (but not GM6001-) and the EGFR inhibitors AG1478 and by PD153035 (**Fig. 5**). Our results, taken collectively, demonstrate that EGFR, MMP/ADAM, and ADAM17 in particular, mediate IL-1beta-dependent Egr-1 expression and IL-1beta-dependent SMC repair after cell injury.

## Discussion

Egr-1 is rapidly expressed upon exposure to a range of pathophysiologic stimuli through mitogen-activated protein (MAP) kinase signaling. Here we now show that IL-1beta induction of Egr-1 is both MMP/ADAM- and EGFR-dependent. Pharmacologic MMP and ADAM17 inhibitors, and ADAM17 siRNA, prevent Egr-1 induction by IL-1beta. Moreover EGFR tyrosine kinase inhibition and EGFR siRNA block IL-1beta of Egr-1. We also report that IL-1beta-inducible SMC repair after injury is both EGFR- and MMP/ADAM(17)-dependent. Cytokine-inducible Egr-1 expression and wound repair is thus a protease-dependent process involving EGFR transactivation.

To our knowledge this is the first report that demonstrates the requirement of MMP/ADAM in cytokine induction of Egr-1. This builds on a recent study with HaCaT cells showing the metalloproteinase inhibitor TAPI-2 inhibits sodium lauryl sulfate (SLS)-induced Egr-1 expression [44]. A previous study examined the link between MMP and Egr-1 expression in A-10 cells responding to the peptide hormone, arginine vasopressin [45]. However treatment with the pan-MMP inhibitor GM6001+ in that study did not inhibit Egr-1 expression but blocked induction of another immediate early gene c-Fos. Although our data demonstrate a role of IL-1beta inducible MMP/ADAM/EGFR-dependent Egr-1 expression, mechanisms by which IL-1beta mediates MMP/ADAM activation still needs clarification. Hall and Blobel have recently shown that in mEFs, IL-1beta stimulates ADAM17 by a mechanism involving p38MAPK but independent of ADAM17 phosphorylation [46].

A well-studied mechanism of EGFR transactivation is GPCR-stimulated shedding of HB-EGF, known as “triple membrane-passing signaling” in which MMPs are responsible for the proteolytic cleavage of the pro-HB-EGF [45]. EGFR ligand shedding is a pre-step required for receptor activation [47] and occurs in response to certain stimuli, such as Ang II, whereby the released ligand initiates intracellular signaling through EGFR [47]. Previous studies have demonstrated EGFR transactivation by IL-1beta in cultured human keratinocytes [48]. Moreover, AG1478 inhibits vasopressin induction of Egr-1 in A-10 SMCs [45] or *H. pylori* activation of Egr-1 in AGS gastric epithelial cells [49]. Future studies should determine whether EGFR ligand shedding accounts for IL-1beta induction of Egr-1.

Egr-1 is an injury-inducible transcription factor [5] that regulates a variety of pro-atherogenic genes which we and others have targeted by various approaches, such as DNAzymes and binding decoys. Egr-1 inhibition perturbs SMC migration and proliferation *in vitro* and intimal thickening in rats [18], rabbits [21] and pigs [20]. It also mediates myocardial inflammation and infarct size after ischemia-reperfusion injury in rats [50] and pigs [51], and regulates angiogenesis and tumor angiogenesis in mice [37]. Egr-1 is therefore an attractive therapeutic target in a range of vascular diseases. The reliance on MMP/ADAM and EGFR of cytokine-inducible Egr-1 expression, as demonstrated here, suggests collateral pathways amplify signals that culminate in Egr-1 expression and opens new opportunities of limiting the expression and activity of this pathophysiologically-important immediate-early gene. Inhibitors of ADAM17 activity or EGFR phosphorylation that decrease Egr-1 expression in SMC could help prevent SMC hyperplasia following coronary angioplasty and stenting.

## Supporting Information

Figure S1 Band intensities for (**A**) Egr-1 protein relative to beta-actin corresponding to Fig. 1B, (**B**) Egr-1 protein relative to beta-actin corresponding to Fig. 1D, and **(C)** ADAM17, Egr-1 and ADAM10 protein relative to beta-actin corresponding to Fig. 1E by scanning densitometry. Figures are representative of at least three independent determinations. Error bars represent mean ± SE.
(TIFF)

Figure S2 Band intensities for (**A**) Egr-1 protein relative to beta-actin corresponding to Fig. 2B. Figures are representative of at least three independent determinations. *p<0.05. Error bars represent mean ± SE. Quiescent SMCs were pretreated with different concentrations of (**B**) AG1478 and **(C)** PD153035 for 30 min, followed by stimulation with IL-1beta (10 ng/ml) for 30 min. Cells were collected and total RNA isolated. cDNA was synthesized and used for real time qPCR analysis. Data were normalized to beta-actin. Band intensities for (**D**) EGFR and Egr-1 protein relative to beta-actin corresponding to Fig. 2C, and (**E**) ErbB4, Egr-1 and EGFR protein relative to beta-actin corresponding to Fig. 2D. Figures are representative of at least three independent determinations. (**F**) Egr-1 protein relative to beta-actin corresponding to Fig. 2F.
(TIFF)

Figure S3 Band intensities for (**A**) EGFR phospho-Tyr^845^ protein relative to total EGFR and Egr-1 protein relative to beta-actin corresponding to Fig. 3A, (**B**) EGFR phospho-Tyr^845^ relative to total EGFR protein corresponding to Fig. 3B, (**C**) EGFR phospho-Tyr^845^ relative to total EGFR protein corresponding to Fig. 3C, and (**D**) EGFR phospho-Tyr^845^ relative to total EGFR protein corresponding to Fig. 3D. Figures are representative of at least three independent determinations. Error bars represent the mean ± SE.
(TIFF)

Figure S4 Band intensities in ADAM17^−/−^ cells and ADAM17WT mEFs for Egr-1, ADAM17, EGFR and IL-1RI protein relative to beta-actin corresponding to Fig. 4A. Figures are representative of at least three independent determinations. Error bars represent the mean ± SE.
(TIFF)
