# Correction: IL-1beta Signals through the EGF Receptor and Activates Egr-1 through MMP-ADAM

**DOI:** 10.1371/annotation/a0bc2cbb-0390-4476-833d-30ce4f829794

**Published:** 2013-12-03

**Authors:** Estella Sanchez-Guerrero, Elya Chen, Maaike Kockx, Si-Wei An, Beng H. Chong, Levon M. Khachigian

There was an inadvertent error in the assembly of panels 'AG1478, IL-1b' and 'PD153035, IL-1b' in Figure 5 of this article; as a result, the panels were duplicated in error. A new figure from separate experiments is provided herewith: http://plosone.org/corrections/pone.0039811.005.cn.pdf

along with the images underlying the new figure:

http://plosone.org/corrections/pone.0039811.g002.cn.tif

http://plosone.org/corrections/pone.0039811.g003.cn.tif

http://plosone.org/corrections/pone.0039811.g004.cn.tif

http://plosone.org/corrections/pone.0039811.g005.cn.tif

http://plosone.org/corrections/pone.0039811.g006.cn.tif

http://plosone.org/corrections/pone.0039811.g007.cn.tif

http://plosone.org/corrections/pone.0039811.g008.cn.tif

http://plosone.org/corrections/pone.0039811.g009.cn.tif

This does not affect the conclusions reported in the article or the original interpretation of the results. The authors apologize for the error. 

