# Supplementary figures and images for: IL-1beta Signals through the EGF Receptor and Activates Egr-1 through MMP-ADAM
Source: PLoS One. 2012 Jul 6;7(7):e39811. doi: 10.1371/journal.pone.0039811 (PMC3391205; doi:10.1371/journal.pone.0039811)

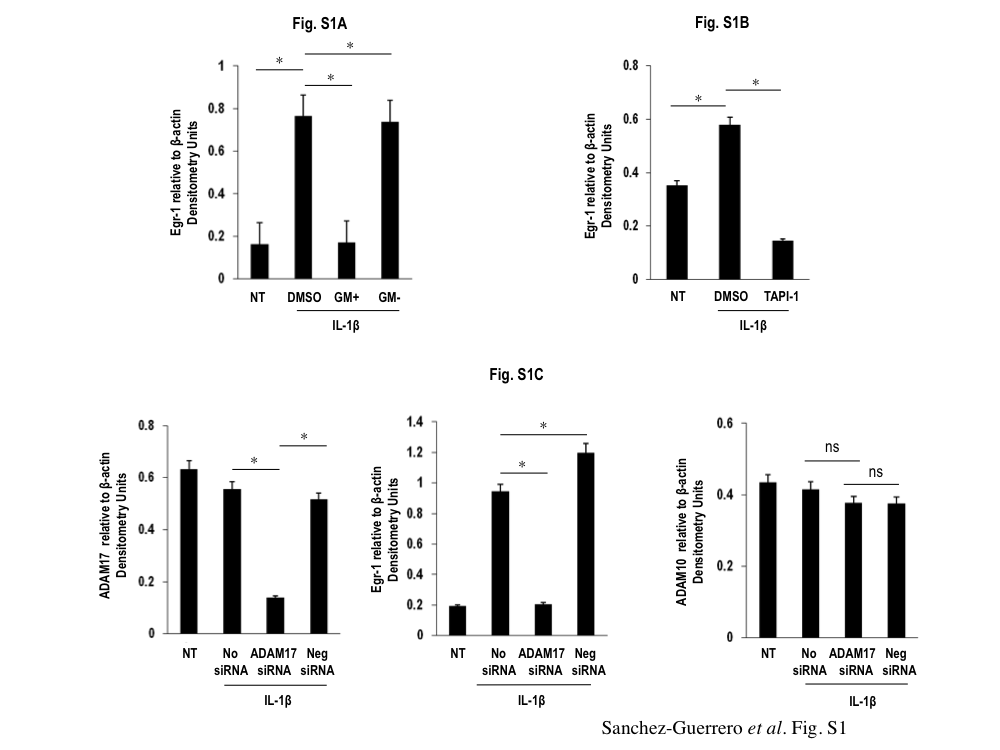

Supplement: Figure S1 — Band intensities for (A) Egr-1 protein relative to beta-actin corresponding to Fig. 1B, (B) Egr-1 protein relative to beta-actin corresponding to Fig. 1D, and (C) ADAM17, Egr-1 and ADAM10 protein relative to beta-actin corresponding to Fig. 1E by scanning densitometry. Figures are representative of at least three independent determinations. Error bars represent mean ± SE. (TIFF) [file pone.0039811.s001.tif]

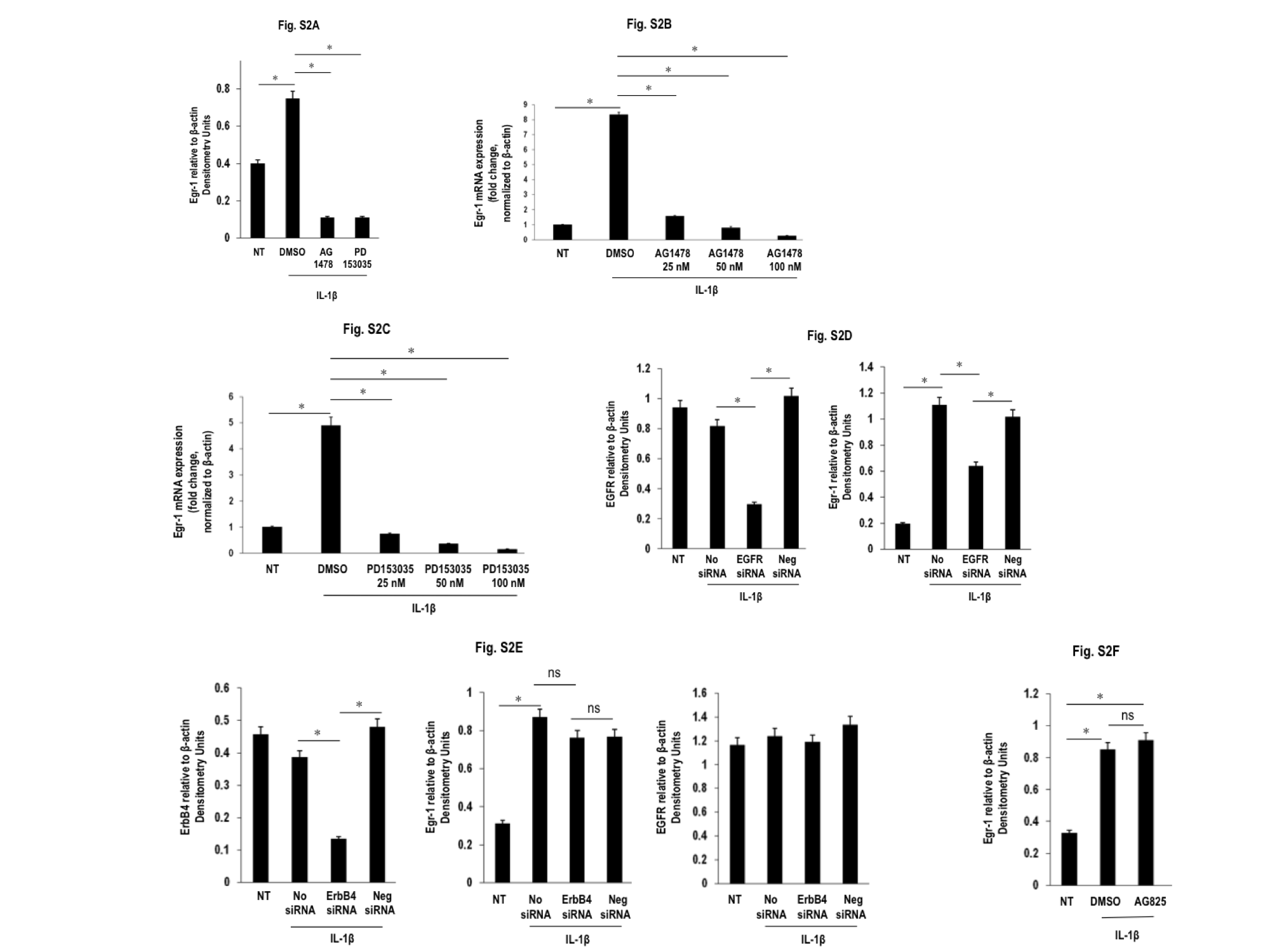

Supplement: Figure S2 — Band intensities for (A) Egr-1 protein relative to beta-actin corresponding to Fig. 2B. Figures are representative of at least three independent determinations. *p<0.05. Error bars represent mean ± SE. Quiescent SMCs were pretreated with different concentrations of (B) AG1478 and (C) PD153035 for 30 min, followed by stimulation with IL-1beta (10 ng/ml) for 30 min. Cells were collected and total RNA isolated. cDNA was synthesized and used for real time qPCR analysis. Data were normalized to beta-actin. Band intensities for (D) EGFR and Egr-1 protein relative to beta-actin corresponding to Fig. 2C, and (E) ErbB4, Egr-1 and EGFR protein relative to beta-actin corresponding to Fig. 2D. Figures are representative of at least three independent determinations. (F) Egr-1 protein relative to beta-actin corresponding to Fig. 2F. (TIFF) [file pone.0039811.s002.tif]

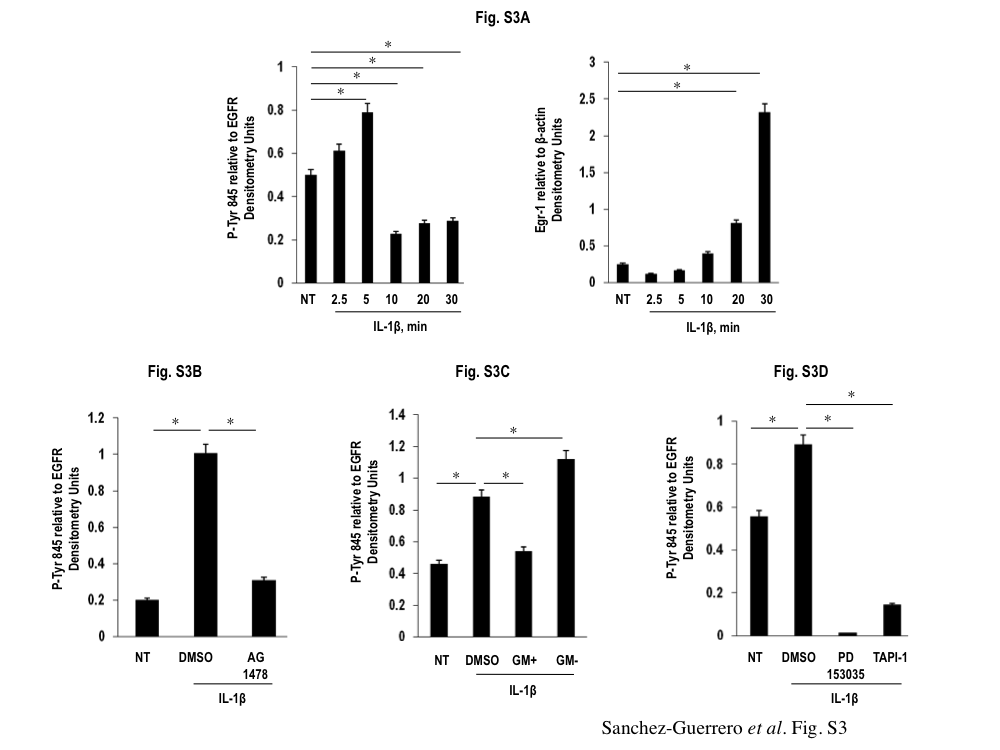

Supplement: Figure S3 — Band intensities for (A) EGFR phospho-Tyr845 protein relative to total EGFR and Egr-1 protein relative to beta-actin corresponding to Fig. 3A, (B) EGFR phospho-Tyr845 relative to total EGFR protein corresponding to Fig. 3B, (C) EGFR phospho-Tyr845 relative to total EGFR protein corresponding to Fig. 3C, and (D) EGFR phospho-Tyr845 relative to total EGFR protein corresponding to Fig. 3D. Figures are representative of at least three independent determinations. Error bars represent the mean ± SE. (TIFF) [file pone.0039811.s003.tif]

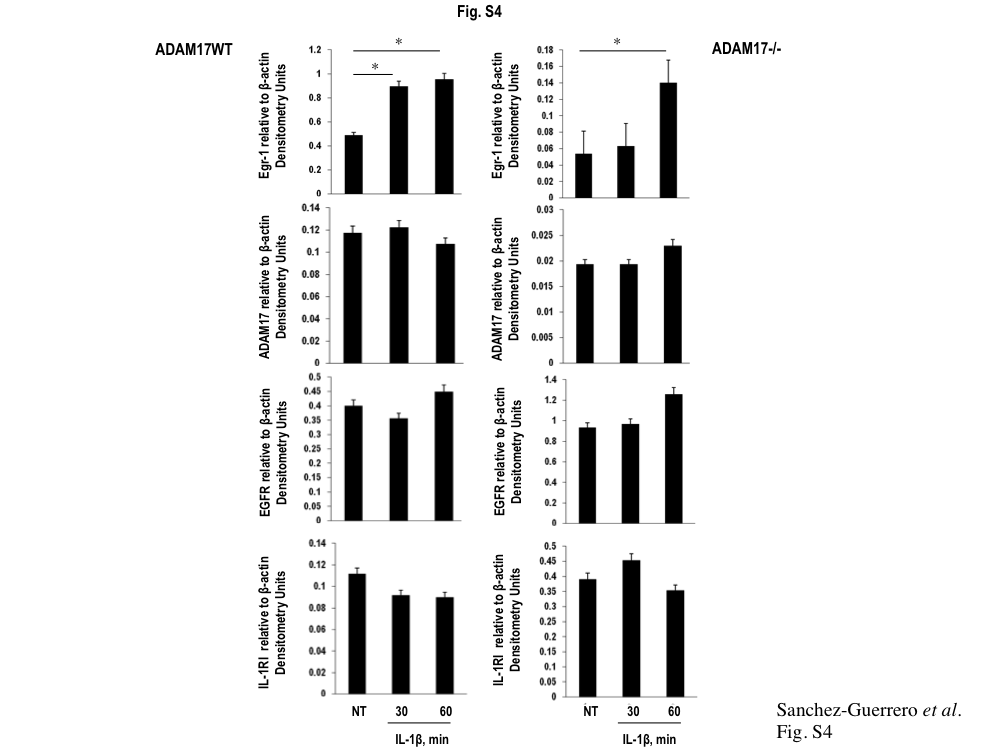

Supplement: Figure S4 — Band intensities in ADAM17−/− cells and ADAM17WT mEFs for Egr-1, ADAM17, EGFR and IL-1RI protein relative to beta-actin corresponding to Fig. 4A. Figures are representative of at least three independent determinations. Error bars represent the mean ± SE. (TIFF) [file pone.0039811.s004.tif]
